# Characterizing Trends in Cancer Patients' Survival Using the JPSurv Software

**DOI:** 10.1158/1055-9965.EPI-21-0423

**Published:** 2021-08-17

**Authors:** Angela B. Mariotto, Fanni Zhang, Dennis W. Buckman, Daniel Miller, Hyunsoon Cho, Eric J. Feuer

**Affiliations:** 1 Division of Cancer Control and Population Sciences, NCI, Bethesda, Maryland.; 2 Information Management Services Inc., Calverton, Maryland.; 3 Department of Cancer Control and Population Health, Graduate School of Cancer Science and Policy, National Cancer Center, Goyang, Gyeonggi-do, Republic of South Korea.; 4 Division of Cancer Registration and Surveillance, National Cancer Center, Goyang, Gyeonggi-do, Republic of South Korea.

## Abstract

**Background:**

Improvements in cancer survival are usually assessed by comparing survival in grouped years of diagnosis. To enhance analyses of survival trends, we present the joinpoint survival model webtool (JPSurv) that analyzes survival data by single year of diagnosis and estimates changes in survival trends and year-over-year trend measures.

**Methods:**

We apply JPSurv to relative survival data for individuals diagnosed with female breast cancer, melanoma cancer, non–Hodgkin lymphoma (NHL), and chronic myeloid leukemia (CML) between 1975 and 2015 in the Surveillance, Epidemiology, and End Results Program. We estimate the number and location of joinpoints and the trend measures and provide interpretation.

**Results:**

In general, relative survival has substantially improved at least since the mid-1990s for all cancer sites. The largest improvements in 5-year relative survival were observed for distant-stage melanoma after 2009, which increased by almost 3 survival percentage points for each subsequent year of diagnosis, followed by CML in 1995–2010, and NHL in 1995–2003. The modeling also showed that for patients diagnosed with CML after 1995 (compared with before), there was a greater decrease in the probability of dying of the disease in the 4th and 5th years after diagnosis compared with the initial years since diagnosis.

**Conclusions:**

The greatest increases in trends for distant melanoma, NHL, and CML coincided with the introduction of novel treatments, demonstrating the value of JPSurv for estimating and interpreting cancer survival trends.

**Impact:**

The JPSurv webtool provides a suite of estimates for analyzing trends in cancer survival that complement traditional descriptive survival analyses.

## Introduction

During the past two decades, substantial progress has been made in the treatment, early detection and prevention of many cancers. Population-based cancer statistics, especially trends in cancer rates (incidence and mortality), are the measures used most frequently to report and monitor progress in cancer control (1, 2). The Joinpoint regression model analyzes rates and proportions over time to identify timepoints (joinpoints) at which trends have changed, and to estimate the regression function with joinpoints identified (3). The Joinpoint regression model also provides a summary of the pace at which rates are changing, usually reported as the annual percent change (APC).

More recently, a Joinpoint survival model (4) was developed that allows for analysis of survival trends by single calendar year of diagnosis. The Joinpoint survival model (4) is an extension of the proportional hazards model for survival, where the effect of year of diagnosis is linear on the log of the probability of cancer death scale (5). Like the Joinpoint model for rates, the Joinpoint survival model estimates the location and number of calendar years where changes in survival trends occurred. However, the Joinpoint survival model has been underutilized in survival studies. Most studies investigating changes in cancer survival simply compare 5-year relative survival of patients with cancer diagnosed in earlier versus later grouped calendar years of diagnosis (6, 7). This type of comparison does not provide information on when or how cancer survival is changing. Reasons for the limited use of the Joinpoint survival model may be related to challenges in model and trend measure interpretation as well as a lack of user-friendly software. Applying the Joinpoint survival model (4) requires knowledge of statistical software packages, for example, SAS or R. Importantly, the trend measure was previously defined as the percent change in the annual probability of cancer death (hazard) scale (4), which is a less used and less intuitive measure of a patient's prognosis.

To make the joinpoint survival analyses more readily available, we developed a JPSurv webtool that can be accessed at https://analysistools.cancer.gov/jpsurv/. The JPSurv webtool is based on the JPSurv R package (https://cran.r-project.org/web/packages/JPSurv/), which uses an iteratively reweighted least squares (IRLS) algorithm to estimate the Joinpoint survival model for relative survival, cause-specific and overall survival. To facilitate the communication of survival trends, we also developed a new measure summarizing trends on the survival scale.

We first present the JPSurv model and the trend measures on the hazard and survival scales. We then briefly describe the JPSurv webtool. We provide an application using relative survival data for patients diagnosed with female breast cancer, melanoma cancer, chronic myeloid leukemia (CML) and non–Hodgkin lymphoma (NHL). These cancer sites were chosen because they illustrate interpretation of survival changes and trends measures for a range of prognoses, and varying dissemination of screening and novel treatments.

## Materials and Methods

The JPSurv software has been developed to analyze survival trends by single year of diagnosis (year-over-year). The Joinpoint survival model (4) is an extension of the proportional hazards model for survival, where the effect of calendar year of diagnosis is linear on the log hazard of cancer death scale. The JPSurv model allows for different linear trends between joinpoints.

### The JPSurv model

We assume that the hazard rate of dying at a time *t* since diagnosis (follow-up) for people diagnosed at calendar year *x*, \lambda (t|x)$ , follows a proportional hazards model




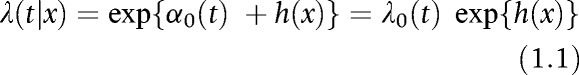




where \lambda _0^{}(t)$ is the baseline hazard and }{}h(x)$ is a joinpoint model, }{}{{h(x) = \beta x\; + \,\sum\limits_{k = 1}^K {{\delta _k}} {(x - {\tau _k})^ + }}} including *K* joinpoints at years \,{\tau _1}, \ldots ,\;{\tau _K} where }{}{u^ + } = u if }{}u > 0 and }{}{u^ + } = 0 otherwise and \beta ,{\delta _1}, \ldots ,{\delta _K}$ the regression coefficients. The slope of the first segment before the joinpoint }{}{\tau _1}$ is \beta $ and the slope of the segment between joinpoints }{}{\tau _k}$ and }{}{\tau _{k + 1}}$ is \beta + \sum\nolimits_{j = 1}^k {{\delta _j}} . When there is no joinpoint, then }{}h(x) = \beta \,x and the model becomes a Cox proportional hazards model with calendar year of diagnosis *x* as a covariate.

### Projecting survival using the JPSurv model

The JPSurv model can also project survival beyond observed data. Thus, suppose data are calendar years 1 to *L*. Survival for patients diagnosed in calendar year *x*, }{}x > L$ , can be estimated as }{}S(t|x) = {S_0}{(t)^{\exp \{ h(x)\} }} , where }{}{S_0}(t) = \exp \{ { - \int_{0}^{t}{{{\lambda _0}(u)\;du}}} \}$ and by extending the last joinpoint segment, that is, calculating }{}h(x) = \beta x\; + \,\sum\limits_{k = 1}^K {{\delta _k}} (x - {\tau _k})\; for }{}x > {\tau _K} . The projection assumes that the trend in the last segment continues in future years. Note that for calendar year *L* usually only 1-year survival, }{}S(1|L) , is observed. Thus, the model also allows for estimation of 2-year, 3-year,…, 5-year survival for patients diagnosed at year *L*.

### Model estimation and model selection

The JPSurv model uses a combination of grid search and IRLS methods (8) to estimate the number of joinpoints, the location of joinpoints, and the regression coefficients of the Joinpoint model. For each number of *k* joinpoints up to a maximum number of 5 joinpoints, we find the model (joinpoint location and regression parameters) that maximizes the likelihood function. Let }{}{M_k}$ denote the estimated *k*-joinpoint model and }{}{l_k}$ its maximum log-likelihood. The Bayesian Information Criterion (BIC) is defined as




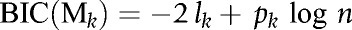




where *n* is the total number of follow-up years for all diagnosis years and }{}{p_k}$ is the number of parameters under the model. The Akaike Information Criterion (AIC) is also provided. Yu and colleagues (4) favored BIC and provided simulations and guidelines on the performance of BIC. In general BIC, had the best performance in selecting the correct model. However, when sample size was small it tended to detect a smaller number of joinpoints. The AIC is more sensitive to changes and estimates a larger number of joinpoints (4). SEs and confidence intervals can be calculated using the delta method as shown in Yu and colleagues (4). Details of the likelihood function and estimation methods for relative survival and cause-specific survival are provided in the Supplementary Materials and Methods.

### Annual percent change in the probability of cancer death (APC_D)

Let \beta^*$ be the slope coefficient in a segment between consecutive joinpoints, so that }{}h(x)\; = \beta^*x , then Yu and colleagues (4) suggested }{}APC\_D = 100\;\{ \exp (\beta^*) - 1\} \% as the annual percent change in the hazard of cancer death for continuous follow-up time. For discrete data, cancer death hazard translate into }{}{\lambda _j}(x) the conditional probability of dying of cancer in interval *j*, being alive at the beginning of the interval, for people diagnosed at year *x*. The conditional probability of cancer death is also called interval or annual probability of cancer death.

When }{}{\lambda _j}(x) is small, we have }{}{{[ {{\lambda _j}(x + 1) - {\lambda _j}(x)} ]} \mathord{/ {\vphantom {{[ {{\lambda _j}(x + 1) - {\lambda _j}(x)} ]} {{\lambda _j}(x)}}} \kern-\nulldelimiterspace} {{\lambda _j}(x)}} \cong \exp (\beta^*)\; - \;1 = {\frac{{APC\_D}}{{100}}} .

Thus, an APC_D = −2% represents an annual percent change of 2% decline in the annual probability of cancer death by single year of diagnosis. It represents a 0.98 annual risk of cancer death for patients diagnosed in a given year compared with those diagnosed in the prior year.

### Annual absolute change in survival (AAC_S)

Prognosis is often reported as cumulative survival, that is, as the probability of being alive at 5 or 10 years after diagnosis. We developed a new measure to summarize survival trends on the cumulative survival scale. For calendar year }{}x \in [{\tau _m},\;{\tau _{m + 1}}] between joinpoints with slope coefficient \beta^* , the cumulative survival probability after *t* years from diagnosis (follow-up), is the product of the interval survival probabilities and can be written as

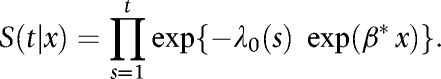


The absolute change in *t*-year survival at calendar year *x* is calculated as

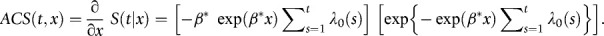


Because it varies by calendar year, we average over all calendar years in the interval. Thus, the (average) annual absolute change over a period of calendar years }{}{x_i} \in [{\tau _m},\;{\tau _{m + 1}}) is estimated as

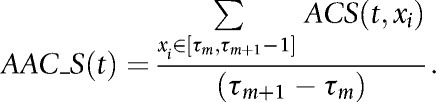


The *AAC_S*(*t*) represents the average difference of cancer survival at *t* years from diagnosis for people diagnosed in a calendar year compared with people diagnosed in the prior year. Since it reflects the difference of 2 percentages, it is measured in percentage points. For example, moving up from 40% to 44% is a 4 percentage point increase in cumulative survival. Table 1 provides comparison and interpretation of APC_D and AAC_S.

**Table 1.** Comparison between measures to summarize trend: annual absolute change in survival (AAC_S) and the annual percent change in the conditional probability of cancer death (APC_D).

Characteristics	**AAC_S: A**nnual **A**bsolute **C**hange in **S**urvival	**APC_D: A**nnual **P**ercent **C**hange in the Conditional Probability of **D**eath
Underlying measure	***S(i,y):* Cumulative survival**, i.e., the probability of surviving cancer after **i** years from diagnosis, for patients diagnosed in year ***y***	***P(i,y):* Conditional probability of dying of cancer in interval *i*** given alive at the beginning of the interval, for patients diagnosed in year ***y***
Formula (approximation)	Average of the survival difference {S(j,y+1)-S(j,y)} for calendar years ***y*** in the joinpoint segment	100*{P(i,y+1)-P(i,y)}/P(i,y) = 100*[exp(B)-1] where B is the coefficient in the joinpoint segment
Unit	**Percentage points (pp):** difference of two percentages	**Percent:** relative change of percentages. Similar to annual percent change (APC) for rates
Examples	Moving from 40% to 42% means a 2–percentage point increase in cumulative survival	Moving from 40% to 38% means a 5 **percent** decrease in the annual probability of cancer death
Interpretation	AAC_S(5) = 2%: The 5-year cancer survival is increasing on average 2 percentage points for each subsequent year of diagnosis.	APC_D = −5%: The annual probability of dying of cancer is decreasing by 5% for each subsequent year of diagnosis, similar to a 0.95 relative risk of dying of cancer in year }{}y + 1 compared with year *y*.
Does it vary by time since diagnosis?	Yes.	No.
	AAC_S(1) ≠ AAC_S(5)	APC_D is the same for 0–1 year, 1- years, … from diagnosis.
Motivation/Summary	More clear prognosis interpretation versus more awkward mathematical derivation.	Clear mathematical interpretation versus challenging prognosis interpretation

The annual absolute change in survival, }{}AAC\_S(t) , depends on follow-up survival time *t*. It can be calculated between any 2 calendar years to estimate the average year-to-year of diagnosis trend in the period, e.g., the most 10 recent calendar years. However, it is usually calculated in a segment between joinpoints. The SE for }{}AAC\_S(t) is obtained using the delta method and is shown in the Supplementary Materials and Methods.

### The JPSurv webtool

JPSurv is web-based tool available at (https://analysistools.cancer.gov/jpsurv/). Input data consist of grouped relative, cause-specific or overall survival data by annual time since diagnosis (follow-up) and by calendar year of diagnosis. Although more granular survival can be of interest for rapidly fatal cancer types, the tool requires annual survival rates and annual calendar year at diagnosis to calculated year-over-year changes in survival. The input data can be imported as a delimited text file or as a SEER*Stat text file and dictionary file calculated using the SEER*Stat software (https://seer.cancer.gov/seerstat/). The following variables are required: survival time interval in annual years since diagnosis, calendar year of diagnosis, number at risk at beginning of interval, number of cases lost to follow-up in the interval, number of cancer deaths (for cause-specific survival), number of deaths (for overall survival), and number of deaths and interval expected survival (for relative survival). Other stratifying variables such as cancer site, sex, stage, etc. which define survival cohorts can also be included. The user has full flexibility to define multiple cohorts by stratifying the survival calculations in SEER*Stat. For example, data may include survival for men and women diagnosed with colorectal and lung cancer. If the user does not select specific values for cancer site or sex, JPSurv will fit models to each combination of sex and cancer site in a single run using the same settings for all cohorts. If the user would like to change the settings for males lung cancer survival, than the user needs to select males and lung cancer, change the settings and run JPSurv for that cohort.

Settings include the maximum number of joinpoints tested (default is 0 joinpoints and maximum is 5), and the diagnosis years to be used in the model estimation (default is all years in the data). For two or more joinpoints or more than one survival cohort computational time is long and the user is required to submit an e-mail address to retrieve results. Advanced options provide control of the minimum number of years required between joinpoints, first and last intervals and the number of years for projections. The JPSurv help (https://analysistools.cancer.gov/jpsurv/html/help.html) and tutorial provide more detailed information on the input data and parameters to be specified by the user.

The default final model is the model with minimum BIC; however, users can use AIC to select their final model and all outputs can be displayed for any of the tested models. The outputs include three types of plots with predicted and observed values as below.

(i) Observed and modeled X-year relative survival (or cause-specific survival; *y*-axis) by calendar year of diagnosis (*x*-axis). The user can select multiple times since diagnosis X to display, for example, 1-year and 5-year.
(ii) X to X+1 annual probabilities of cancer death (*y*-axis) by calendar of diagnosis (*x*-axis). The user can select multiple times since diagnosis X to display.
(iii) Cumulative relative survival or cause-specific survival (*y*-axis) by time since diagnosis (*x*-axis) for people diagnosed in year Y. The user can select multiple calendar year of diagnosis and display for example, three survival curves by time since diagnosis for patients diagnosed in 1990, 1995, and 2000.

The graphs allow for some customization. Trend measures are also calculated and can be displayed in the plots.

### Application to the SEER data

We used data from the Surveillance, Epidemiology and End Results (SEER) program November 2019 data submission. We calculated relative survival for patients with cancer diagnosed between 1975–2015 and followed through 2017 in the SEER-9 areas using the SEER*Stat software (9). We selected patients diagnosed with female breast and melanoma cancers by historic stage (local, regional, and distant) at diagnosis (10) and NHL and CML. Because of changes in staging codes, historic stage is only available through cases diagnosed in 2015. We excluded cases diagnosed by autopsy or death certificate and those lost to follow-up in the month of diagnosis (alive and no survival time). Although patients diagnosed many years ago have more than 10 years of follow-up, we only included relative survival for a maximum of 10 years of follow-up. The choice of follow-up time to include in the modeling depends on the interest in understanding longer survival. If only trends in 5-year survival will be reported, the user should only include up to 5-years of follow-up. We used the JPSurv software with the default parameters and tested a maximum number of 4 joinpoint for all the cancer sites and stage combinations with the exception NHL for which we used 5 joinpoints. The cancer sites in this study were chosen because of the introduction of new treatments that are known to have improved survival. We report models with the minimum BIC.

Only for CML and to check the proportionality assumption we performed analyses limiting follow-up time to up to 2 and 5 years.

## Results

### Overview of measures and interpretation

**Figure 1.**
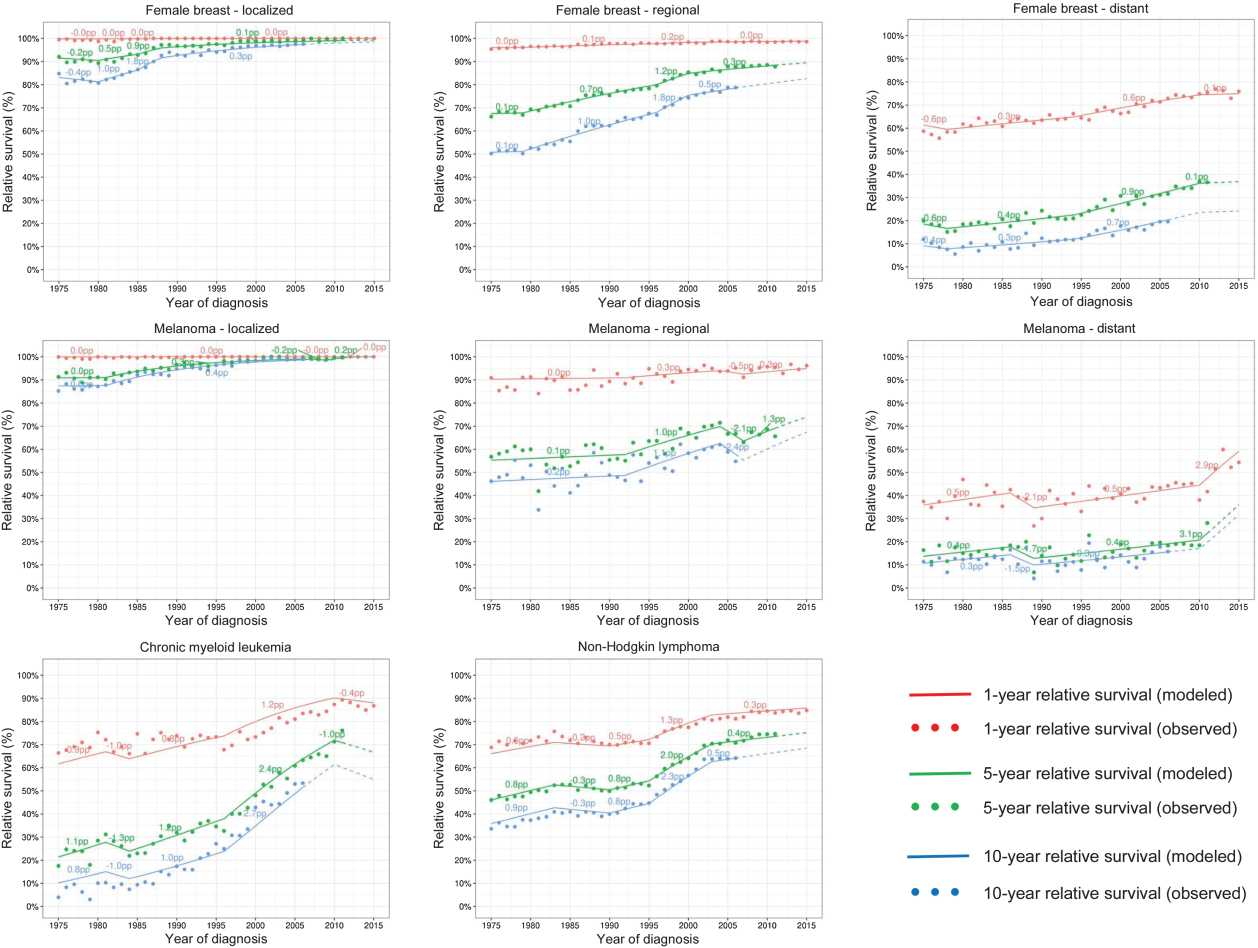
Observed (dots) and modeled (lines) 1-year, 5-year, and 10-year relative survival by year of diagnosis. Female breast and melanoma cancers are shown by stage at diagnosis while CML and NHL include all stages. The numbers above the lines correspond to the average absolute change in survival (AAC_S) trend measure in between joinpoints. Dashed lines represent modeled projected survival.

Relative survival has substantially improved since at least the mid-1990s for all combinations of cancer sites and stages (Fig. 1). The numbers near the lines represent the AAC_S between joinpoints for the 1-year, 5-year, and 10-year relative survival. Visually the largest improvements occurred for CML and NHL after the mid-1990s and distant melanoma after 2010. Supplementary Table S1 displays the BIC and AIC measures for all the fitted models including the chosen final model with the minimum BIC.

The trend measures and their respective 95% confidence intervals for the final models are displayed in Table 2. The greatest increases in 5-year relative survival were observed for distant melanoma after 2009 [AAC_S(5) = 2.86 pp], CML in 1995–2010 [AAC_S(5) = 2.48 pp], and NHL in 1995–2003 [AAC_S(5) = 2.00 pp]. This means that for CML, 5-year relative survival increased approximately 2.5 percentage points (pp) for each subsequent year of diagnosis representing an increase of 37 = 2.48*15 percentage points in survival in 15 years. This estimated increase corresponds to the observed increase in 5-year relative survival from 35% in 1995 to 71% in 2010. The AAC_S(1) in 1-year survival provides a similar ranking in trends as the AAC_S(5) but at a smaller level of increase.

**Table 2.** Number and location of joinpoints in the final model and trend measures: annual absolute change in 5-year and 1-year AAC_S(5) and AAC_S(1), respectively, and percent change in the annual probability of cancer death (APC_D) with respective 95% confidence intervals.

Cancer site	Stage	No. JPs final model	No. alive	Start year	End year	AAC_S(5)	AAC_S(5) 95% C.I.		AAC_S(1)	AAC_S(1) 95% C.I.		APC_D	APC_D 95% C.I.	
Breast	Local	2	4,163	1975	1982	−0.05 pp	(−0.226 to 0.126)	n.s.	0.00 pp	(−0.005 to 0.003)	n.s.	0.6%	(−1.485 to 2.662)	n.s.
				1982	1988	0.81 pp	(0.669–0.951)	↑	0.02 pp	(0.001–0.031)	↑	−**12.3%**	(−14.184 to −10.371)	↓
				1988	2015	0.13 pp	(0.116–0.151)	↑	0.00 pp	(0.000–0.005)	↑	−**6.7%**	(−7.652 to −5.782)	↓
Breast	Regional	3	3,363	1975	1979	0.07 pp	(−0.285 to 0.424)	n.s.	0.01 pp	(−0.042 to 0.062)	n.s.	−0.3%	(−1.594 to 1.069)	n.s.
				1979	1996	0.72 pp	(0.666–0.773)	↑	0.10 pp	(0.089–0.106)	↑	−3.2%	(−3.462 to −2.965)	↓
				1996	2000	**1.23 pp**	(1.047–1.416)	↑	0.15 pp	(0.128–0.174)	↑	−**7.5%**	(−8.580 to −6.396)	↓
				2000	2015	0.31 pp	(0.247–0.365)	↑	0.04 pp	(0.029–0.043)	↑	−2.6%	(−3.051 to −2.050)	↓
Breast	Distant	1	676	1975	1993	0.30 pp	(0.212–0.391)	↑	0.28 pp	(0.197–0.370)	↑	−0.9%	(−1.234 to −0.665)	↓
				1993	2015	0.79 pp	(0.711–0.865)	↑	0.54 pp	(0.486–0.592)	↑	−2.2%	(−2.391 to −1.962)	↓
Melanoma	Local	1	941	1975	1981	0.02 pp	(−0.307 to 0.354)	n.s.	0.00 pp	(−0.002 to 0.002)	n.s.	−0.3%	(−4.083 to 3.538)	n.s.
				1981	2015	0.25 pp	(0.223–0.277)	↑	0.00 pp	(−0.004 to 0.008)	n.s.	−**8.7%**	(−9.715 to −7.760)	↓
Melanoma	Regional	0	134	1975	2015	0.47 pp	(0.380–0.559)	↑	0.12 pp	(0.096–0.147)	↑	−1.6%	(−1.884 to −1.292)	↓
Melanoma	Distant	1	60	1975	2009	0.14 pp	(0.046–0.237)	↑	0.18 pp	(0.056–0.297)	↑	−0.5%	(−0.808 to −0.153)	↓
				2009	2015	**2.86** pp	(2.096–3.629)	↑	**2.81** pp	(2.103–3.517)	↑	−**7.9%**	(−9.876 to −5.885)	↓
CML	All	2	294	1975	1995	0.67 pp	(0.453–0.879)	↑	0.50 pp	(0.338–0.671)	↑	−1.9%	(−2.464 to −1.266)	↓
				1995	2010	**2.48** pp	(2.208–2.743)	↑	**1.23** pp	(1.079–1.371)	↑	−**7.5%**	(−8.357 to −6.578)	↓
				2010	2015	−1.05 pp	(−2.479 to 0.375)	n.s.	−0.42 pp	(−1.009 to 0.160)	n.s.	4.2%	(−1.428 to 9.897)	n.s.
NHL	All	4	1,731	1975	1983	0.82 pp	(0.455–1.177)	↑	0.61 pp	(0.339–0.873)	↑	−2.3%	(−3.311 to −1.322)	↓
				1983	1990	−0.30 pp	(−0.625 to 0.017)	n.s.	−0.22 pp	(−0.454 to 0.011)	n.s.	0.9%	(−0.051 to 1.841)	n.s.
				1990	1995	0.76 pp	(0.356–1.165)	↑	0.55 pp	(0.257–0.841)	↑	−2.2%	(−3.387 to −1.055)	↓
				1995	2003	**2.00** pp	(1.785–2.216)	↑	**1.33** pp	(1.188–1.481)	↑	−**6.6%**	(−7.327 to −5.949)	↓
				2003	2015	0.40 pp	(0.266–0.542)	↑	0.25 pp	(0.165–0.336)	↑	−1.7%	(−2.324 to −1.137)	↓

Note: Arrows up represent increasing survival; arrows down represent decreasing survival trends and n.s. are not statistically significant trends either increasing or decreasing. Bold numbers represent AAC_S(5) and AAC_S(1) > 1 survival percentage point (pp) for a later year of diagnosis, and APC_D<−5%.

The trend measures in the survival (AAC_S) and in the annual probability of death (APC_D) scales have opposite signs (Table 2). When AAC_S is positive and survival is increasing, then APC_D is negative and the annual probability of dying of cancer is decreasing. In general, AAC_S and APC_D, provided consistent indication if the trends were statistically significant or not significant. No significant changes in survival trends or in the annual probabilities of cancer deaths were observed for local and regional breast cancer, local melanoma cancers in the late 1970s, for CML after 2010, or NHL in 1983–1990. The 1-year AAC_S was also non-significant for local-stage melanoma between 1981 and 2015.

**Figure 2.**
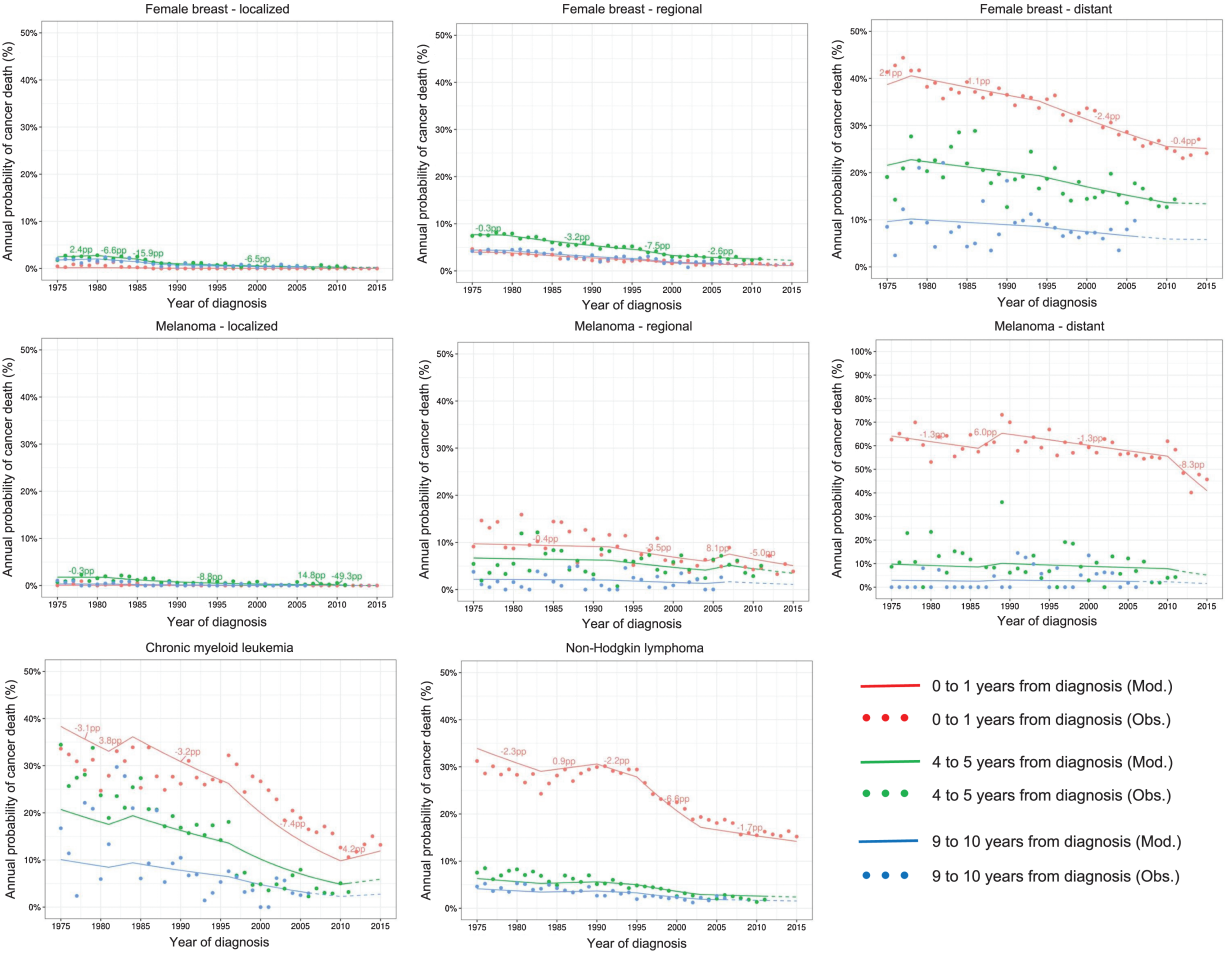
Observed (dots) and modeled (lines) conditional probabilities of dying of cancer in the 0–1, 4–5, and 9–10 interval since diagnosis for those alive at the beginning of the interval, by year of diagnosis. Female breast and melanoma cancers are shown by stage at diagnosis while CML and NHL include all stages. The numbers above the lines correspond to the percent change in the probabilities of cancer death (APC_D) trend measure in between joinpoints. Dashed lines represent modeled projected survival.

However, the AAC_S and APC_D measures differ in ranking the trends. The largest improvement in 5-year relative survival was observed for distant stage melanoma cancer after 2009, while the largest decline in the annual probability of cancer death was observed for local-stage breast cancer between 1982 and 1988. Because APC_D is a percent change (relative measure), it depends on the level of the probability of death. It reports the greatest change when the probability of death is small, and the prognosis is good. Figure 2 shows annual probabilities of cancer death and the respective APC_D measures on a variable *y*-axis scale. For local- and regional-stage breast and melanoma cancer, the annual probabilities of dying of cancer are very small and it is challenging to plot using the 0 to 100 scale.

### Model fit and the proportionality assumption: CML example

**Figure 3.**
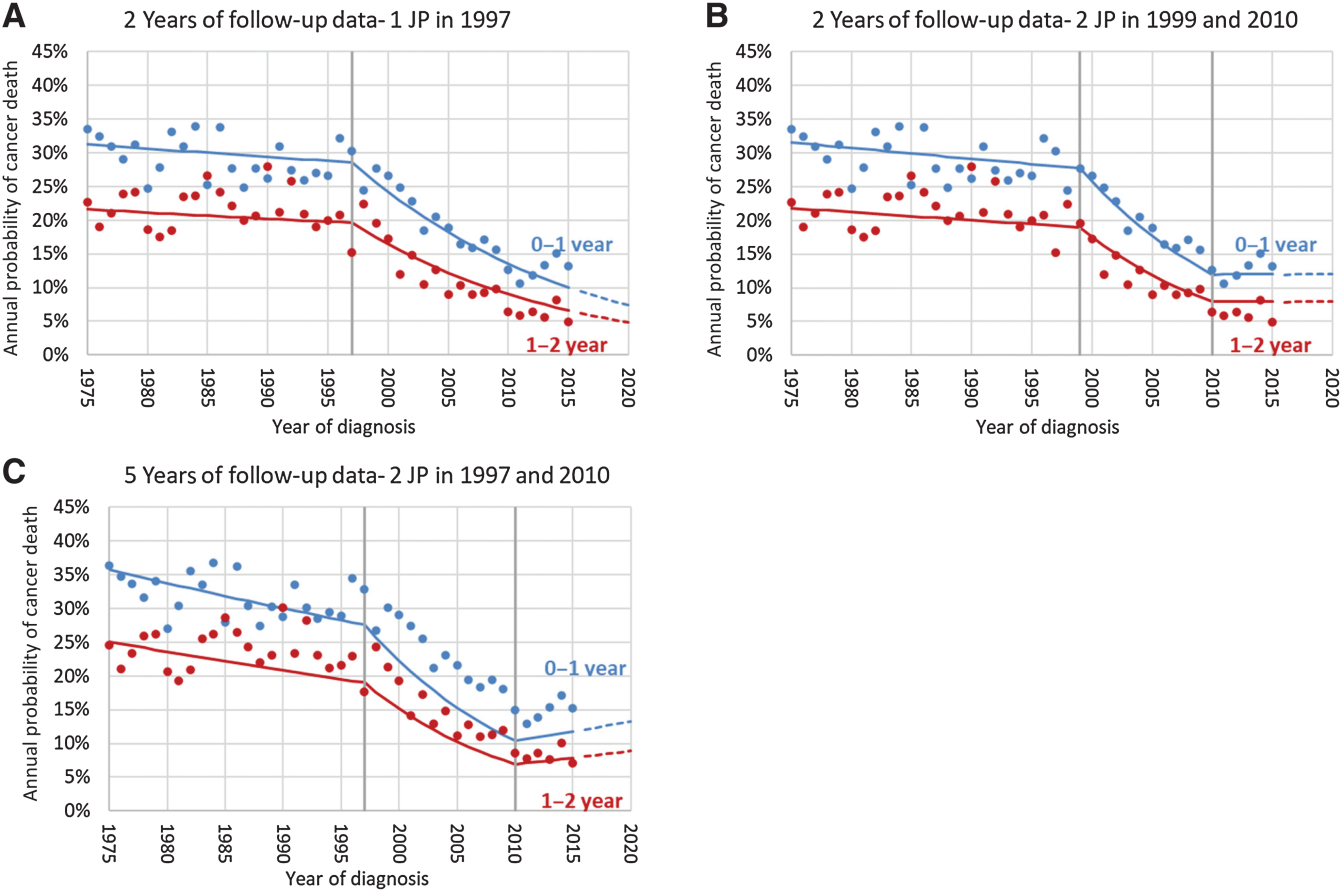
Observed (dots) and modeled (lines) annual probabilities of dying of cancer in the 0–1 and 1–2 annual intervals since diagnosis by year of diagnosis, for patients diagnosed with CML. The figures correspond to models using up to 2 years of follow-up (**A** and **B**) and 5 years of follow-up (**C**). **B** is the final model using the BIC criteria while **A** is the final model using the AIC criteria. The dashed lines are projections beyond available data. JP, joinpoints.

The joinpoint model did not fit well the CML survival data (Fig. 1 and Fig. 2). The lack of fit, is more clearly understood from the annual probability of cancer death figures (Fig. 2). The probability of cancer death in the first year after diagnosis is underestimated and overestimated before and after 1995, respectively. The reverse occurs for patients who survive 4 years: the probability of cancer death in the 5th year after diagnosis is overestimated and underestimated before and after 1995, respectively. To check on the proportionality assumption, we performed analyses limiting follow-up time to up to 2 and 5 years. Using follow-up data up to 2 and 5 years, the BIC criteria identified respectively a model with 1 joinpoint in 1997 and 2 joinpoints in 1999 and 2010, (Table 3; Fig. 3). Figure 3 shows that the fit of the observed data to the 1-year annual probability of death between 2000–2010 is improved when using only 2 years of follow-up. The lack of fit using longer follow-up is because the baseline hazard of cancer death did not decrease proportionally before and after 1997–1999, which roughly coincides with the introduction of imatinib in 2000 to treat CML. In other words, the lack of proportionality shows that although the probability of dying of CML decreased for all intervals after diagnosis, it decreased more for patients surviving 3 or more years from diagnosis in the period after the introduction of imatinib compared with the period before. The AIC criteria identified 2 joinpoints in 1999 and 2010 using only 2 years of follow-up, which provides a joinpoint closer to the introduction of imatinib and better projections. Using 5 years the AIC and BIC select the same model (Table 3).

**Table 3.** Model fit for CML using up to 2 years and 5 years of follow-up data to fit the JPSurv model.

Cancer site	Max follow-up	No. of JPs	Loc. of JPs	BIC	AIC	Log likelihood	Converged	Final model BIC
CML	2 years	0		256.25	231.77	−112.89	Yes	No
		1	1997	**138.02**	105.39	−48.69	Yes	Yes
		2	1999, 2010	140.34	**99.55**	−44.77	Yes	No
		3	1999, 2003, 2010	149.60	100.64	−44.32	Yes	No
		4	1980, 1983, 1999, 2010	155.06	97.95	−41.97	Yes	No
CML	5 years	0		619.73	567.15	−277.58	Yes	No
		1	1996	482.62	421.27	−203.64	Yes	No
		2	1997, 2010	**473.03**	**402.92**	−193.46	Yes	Yes
		3	1985, 1997, 2010	482.33	403.46	−192.73	Yes	No
		4	1981, 1984, 1998, 2010	485.95	398.32	−189.16	Yes	No

Note: Fit measures include the BIC, the AIC, and log-likelihood function. The BIC and AIC used to select the final models are displayed in bold.

## Discussion

### JPSurv webtool and trend measures interpretation

We developed a user-friendly webtool that makes analyses of survival trends more broadly available without the need for any programming skills. We introduced a new measure on the cumulative survival scale, the AAC_S, which facilitates interpretation and comparisons of survival trends. The AAC_S measure represents an average annual absolute change in survival percentage points for people diagnosed in 1 calendar year compared with the prior year. It better reflects the most frequently reported survival statistics, that is, 5-year or 10-year cumulative survival. Because it is an absolute measure, starting with a 5-year survival of 50% in 1980, an increase of 1 percent point, means that 5-year survival for people diagnosed 10 years later will be 60%. However, AAC_S depends on follow-up time (years since diagnosis) and estimates different values for 1-year versus 5-year cumulative survival in the same segment of the joinpoint model. The original measure, APC_D, is like the commonly used APC for rates; however, it is measured at the hazard or probability of cancer death scale. It reflects percent change in the annual probabilities of cancer death for a person diagnosed a year later (4). The main advantages of the APC_D are: (i) it does not depend on follow-up time, (ii) it is constant within a joinpoint segment, and (iii) it can provide insights into the fitness of the JPSurv model and on the proportionality assumption of the baseline hazard of death.

### Implication of the SEER survival trend results

In applying JPSurv to the SEER data, we have considered cancer sites that were impacted by the introduction of novel treatment and screening (breast and melanoma). Survival increased for all cancer sites and all stage combinations. The largest improvement in survival occurred for distant-stage melanoma cancer after 2009, with an increase of 2.8 percentage points in 1-year relative survival for each subsequent year after diagnosis. This improvement coincides with the rapid dissemination of immune therapy for the treatment of advanced melanoma after 2011 (11–14).

The second largest improvement in relative survival was observed for CML between 1995 and 2010. However, the Joinpoint survival model did not fit well the data. The observed survival data violated the baseline hazard proportionality assumption and did not decrease proportionally for all follow-up times across all years of diagnosis. Thus, the trend measure may not be accurate. The first tyrosine kinase inhibitor (TKI) to treat patients with CML was approved by the FDA in 2000 (15, 16). Restricting the data to 2 years of follow-up, improved the fit of 1-year and 2-year survival and identified a joinpoint in 1997 using the BIC criteria and 2 joinpoints in 1999 (closer to 2000) and 2010 using the AIC criteria. The lack of fit indicated that after 2000 there were larger improvements in the chances of surviving CML for patients who had already survived 3 years or more compared with the initial years after diagnosis. This may indicate that patients with CML treated with TKIs who survive 2 or 3 years, subsequently experience a lower hazard of death and were in remission for a long time compared with patients diagnosed in the pre-TKI era.

Relative survival for NHL also increased substantially, 2.0 survival percentage points between 1995 and 2003, coinciding with the dissemination of CHOP (cyclophosphamide, doxorubicin, vincristine, and prednisone) therapy in 1993. This chemotherapy regimen, not only induced remissions in most patients with aggressive NHL, but it also “cured” a significant number of patients with some subtypes of NHL (17).

### Challenges and opportunities

One important limitation of the JPSurv model is the proportionality assumption of the baseline hazard by time since diagnosis for different calendar years of diagnosis. For most applications and in all of the examples except CML, the proportionality assumption was reasonable (18). It assumes that a new treatment decreases the baseline hazard rate proportionally compared with a year in which conventional treatment was given. For CML this was not true. The observed relative risks of dying of cancer in the first year since diagnosis compared with the fifth year was on average 2 prior to the introduction of imatinib and became 4 after 2000, indicating a larger improvement for patients surviving 3 or more years. Relaxing the proportionality assumption adds complexity to the estimation and interpretation. The figure displaying the probability of death for different time since diagnosis can provide a visual check for the proportionality assumption. We are developing extensions of the JPSurv model that will relax the proportionality assumption. In the case of nonproportional data, the JPSurv model can still be applied to survival data with limited follow-up time and also provide insights into how treatment has differentially improved survival by time since diagnosis, as shown in the CML example.

The examples show that JPSurv can be helpful for understanding the impact of cancer treatment advances at the population level, especially for cancer sites unaffected by early detection or screening, such as: NHL, CML, and distant-stage melanoma. However, screening and early detection can introduce biases that artificially increase survival, which complicate interpretation. Screening can (i) advance the time of diagnosis (lead time bias); (ii) include a higher proportion of slower growing cancers, which are most likely to be picked up by screening (length bias); and (iii) detect slow growing cancers that would never cause symptoms or death (overdiagnosis). In the presence of screening, previous work (19, 20) recommends analyzing changes in survival in conjunction with incidence and mortality trends. Thus, it is possible that the improvements in localized breast and melanoma cancers were attributable to screening biases rather than to real improvements in treatment.

The JPSurv model can also be used to predict survival beyond the observed data, both in future calendar years as well as time since diagnosis. Cancer patients' survival projections are useful to for estimating survival among more recently diagnosed patients, in simulation studies, and in prevalence projections to study sensitivity to different future survival projection assumptions. Previous work (21) has shown that extrapolating linear survival trends provides in most cases accurate survival predictions for up to 4 future years. However, caution should be used when extrapolating survival for longer number of years and when the last joinpoint is close to the last year of data. The BIC criteria, used as JPSurv default, is more parsimonious than the AIC and log-likelihood measures. However, users have flexibility to use less parsimonious models.

In summary, the JPSurv webtool provides a suite of estimates and graphs for analyzing cancer survival trends that complement traditional descriptive approaches. Our hope is that JPSurv will improve the reporting of cancer survival trends and interpretation. However, caution should still be used when interpreting survival trends for those cancer sites for which screening or early detection have been widely disseminated in the population, because survival increases may reflect biases rather than real improvements.

## Authors’ Disclosures

No disclosures were reported.
